# The Impact of Unbalanced Maternal Nutritional Intakes on Oocyte Mitochondrial Activity: Implications for Reproductive Function

**DOI:** 10.3390/antiox10010091

**Published:** 2021-01-11

**Authors:** Gemma Fabozzi, Benedetta Iussig, Danilo Cimadomo, Alberto Vaiarelli, Roberta Maggiulli, Nicolò Ubaldi, Filippo Maria Ubaldi, Laura Rienzi

**Affiliations:** 1 Clinica Valle Giulia, GeneraLife IVF Centers, 00197 Rome, Italy; cimadomo@generaroma.it (D.C.); alberto.vaiarelli@gmail.com (A.V.); maggiulli@generaroma.it (R.M.); ubaldi.fm@gmail.com (F.M.U.); rienzi@generaroma.it (L.R.); 2 Genera Veneto, GeneraLife IVF Centers, 36063 Marostica, Italy; iussig@generaveneto.it; 3 Catholic University of the Sacred Heart, 20123 Rome, Italy; ubaldi.nicolo@gmail.com

**Keywords:** diet, nutrition, mitochondria, oocytes, reproductive competence, developmental

## Abstract

Accumulating evidence on the effect of nutrition on reproduction is emerging from both animal and human studies. A healthy dietary pattern and nutrient supplementation, especially during the peri-conceptional period, might be helpful to achieve a live birth, although the mechanisms implicated are not fully understood. The endocrine system and the ooplasmic organelles apparatus, in particular the mitochondria, are clearly key elements during oogenesis and subsequent embryo development, and their proper functioning is associated with nutrition, even beyond maternal aging. Several studies in animal models have reported various adverse effects on mitochondria caused by unbalanced dietary intakes such as high fat diet, high fat high sugar diet, and low protein diet. The alterations produced might include mitochondrial intracellular distribution, content, structure, biogenesis, and functioning. This review summarizes the key role of mitochondria in female reproduction and the effects of different dietary macronutrient compositions on oocyte mitochondrial activity with their possible short-, medium-, and long-term effects.

## 1. Introduction

Maternal diet might harm women’s fertility, as suggested by several emerging evidence. Obese women, for instance, show reduced chance of conceiving both spontaneously and after medically assisted reproduction (MAR). However, obesity is just the tip of an iceberg that entails ovulatory subfertility, anovulatory infertility, and increased miscarriage rates after implantation. During MAR treatments, obese women show decreased responsiveness to gonadotrophins, retrieve fewer oocytes and of poorer quality, display reduced blastulation rates, and increased miscarriage rates [1]. In general, the maternal nutritional intake might affect the physiology of the mechanisms required for the acquisition of oocyte developmental competence during oogenesis. In fact, although female gametogenesis is a long path initiated in fetal life, its last crucial steps occur cyclically after menarche. It is exactly in this peri-conceptional period that nutrition might cause short-, medium-, and long-term effects on reproduction and on the health of the offspring. All these adverse outcomes have been imputed especially to epigenetic modifications and mitochondrial impairments [2,3,4]. 

During oogenesis, human oocytes remain arrested at the diplotene stage of the first meiotic division (germinal vesicle [GV] stage) within their follicles until they are stimulated to undergo a series of fast processes ultimately yielding the ovulation of a mature metaphase II (MII) oocyte [5]. Cyclically, several primordial follicles are recruited to grow until a dominant follicle completes its maturation. Meanwhile in the oocyte, a massive intracellular synthesis of RNA and proteins occurs in order to store as much material as possible for sustaining future embryo development until embryonic genome activation (EGA), which in humans occurs in the transition between the four to the eight cell stage [6]. Follicle morphology changes and increases in size since (i) the flattened pre-granulosa cells become cuboidal, (ii) water, lipids, carbohydrates, and ions are accumulated, and (iii) cytoplasmic organelles like mitochondria are replicated [5]. At the same time, oocyte maturation is attained with two simultaneous events: nuclear (i.e., GV to MII transition) and cytoplasmic maturation, the latter of which involves the synthesis and relocation of organelles like mitochondria, and cumulus expansion/mucification, in turn converting granulosa cells from a tightly packed cellular mass to a more diffuse and dispersed mucified mass [7,8]. All these events are regulated by a plethora of molecules, clearly modulated by the nutritional intake (pyruvate, fatty acids, inorganic ions, second messengers, cytokines, growth factors, etcetera), in a choreography that also requires cooperation between the granulosa cells and the follicular fluid [9]. Remarkably, defective metabolism of glucose and fatty-acids at any phase of oogenesis have been associated with either impaired oocyte developmental competence or future embryo/fetal development [10,11,12]. From a broader perspective, nutrition can also affect the release of specific cytokines into the blood circulation, which in turn might impact the endocrine system and different tissues including the ovary itself [1,13], with direct consequences on the whole apparatus of organelles in the ooplasm (mitochondria, endoplasmic reticulum, and Golgi complex) [14]. In this scenario, amongst the short- and medium-term negative outcomes, nutrition can affect ovulation, oocyte quality, and blastocyst development; whereas, among the long-term effects there are fetal development of organs, cardiovascular effects, muscle biology, offspring fertility, behavioral effects [14].

In this review we focused on the putative effects of different maternal nutritional intakes on mitochondria, possibly the most important organelles responsible for the acquisition/maintenance of oocyte and embryo competence [15,16].

## 2. Search Methods

The PubMed database was searched up to the 31 October 2020 with no time limits through the keywords “maternal nutrition” OR “diet” AND “mitochondria” AND “oocyte”. 

The search produced 35 results that were carefully read and scrutinized for their relevance to the topic under investigation. Only articles with the full text available in English were included. Studies not relevant to the topic under investigation (for example, not focused on maternal peri-conception nutrition or referring only to the effects on embryos or on mitochondria in tissues other than the ovary) were not included. A total of 10 original articles were included in this narrative review (listed in Table 1 and described in detail in this manuscript). Additional references for the introduction and discussion were collected from the most relevant articles among the former 35.

## 3. Brief Overview of the Mitochondria

**Structure and role in cell functioning.** Mitochondria are maternally-inherited intracytoplasmic organelles with a diameter <1.5 μm and a length of 2–8 μm. They own a separate genome (mitochondrial DNA, mtDNA) replicating independently of the cell cycle, consisting of a double-stranded circular histone-free DNA molecule including 37 genes without introns. Mitochondria are enclosed within two highly specialized membranes, separated by a narrow intermembrane space. The outer membrane is smooth and has a protective function, while it offers little resistance to the flow of substances entering and exiting the mitochondrion. Immediately below there is an internal membrane, which folds several times on itself, giving rise to structures called “cristae” to increase the area of their surface. The mitochondrial matrix is located within the inner membrane and hosts the mitochondrial genome

Mitochondria are considered as the powerhouse of cells, providing them with the energy for chemical reactions, namely ATP (adenosine triphosphate), via a biochemical process called oxidative phosphorylation (OXPHOS). Although pyruvate is the primary metabolic substrate used by mitochondria, fatty acids can also be used as a source of acetyl-CoA after undergoing β-oxidation. Briefly, pyruvate generated from glucose through glycolysis in cell cytosol crosses the mitochondrial membranes via monocarboxylate transporters (MCTs) and reaches the mitochondrial matrix where it is metabolized by the pyruvate dehydrogenase complex to produce acetyl-CoA. Acetyl-CoA is in turn oxidized via the Krebs cycle, which generates NADH and FADH_2_, then enters OXPHOS in the inner mitochondrial membrane. The electrons produced by the reduction of these co-factors are transferred to the electron transport chain (ETC) located in the inner mitochondrial membrane, thus allowing the activation of the pumping of protons (H+) in the intermembrane space against their gradient; the final acceptor of electrons is oxygen, ultimately reduced to water. The proton gradient is subsequently exploited to activate the transmembrane enzyme ATP-synthase, which is able to convert ADP to ATP, the chemical compound that provides the cell with the energy needed to perform any kind of biological process.

Beyond ATP production, mitochondria are also involved in calcium buffering, reduction-oxidation (REDOX) homeostasis, synthesis of fatty acids, amino acids, nucleotides, initiators and transducers of signals through the modulation of metabolite availability, reactive oxygen species (ROS) production, regulation of chromosome segregation, stem cell differentiation, and apoptosis [4].

**Oxidative stress: cytokine and chemokine signaling.** Although mitochondrial respiration is a highly controlled process, adverse events may occur. For instance, during pyruvate oxidation, excessive electron leakage from the ETC can occur, leading to a partial reduction of O_2_ to superoxide anion (O_2_^−^), a by-product that can generate ROS, like hydrogen peroxide (H_2_O_2_). ROS can pass through the cell membranes and alter lipids, proteins, and nucleic acids. The mechanisms leading to ROS production in mitochondria are different and have been comprehensively summarized by Cobley [17]. Of note, while on the one hand physiological ROS levels are required to allow the correct biological processes like meiotic resumption [17,18,19], on the other hand a higher production leads instead to both cellular and sub-cellular issues. In oocytes, ROS (i) affect spindle organization and chromosome alignment [20]; (ii) induce endoplasmic reticulum stress and dysfunction of the proteasome causing defective Ca^2+^ homeostasis, impaired protein folding, and eventually apoptosis [21]; (iii) reduce mitophagy, namely the autophagy of defective mitochondria [21]; (iv) affect telomeres [21]; and (v) affect mtDNA integrity [22], which is particularly sensitive to stressors since it does not possess protective histones and efficient DNA repair mechanisms, resulting in a mutation rate 25-times higher than genomic DNA [23].

Beyond endogenous factors like O_2_^−^ generation, exogenous factors can also enhance the production of ROS within oocytes and embryos, for instance lifestyle habits like smoking, excessive caffeine, alcohol, stress, sport, drug abuse, chronic exposure to environmental pollutants, oxygen tension, and light during in vitro culture, and diet [24]. In particular, nutrition is key for balancing antioxidants and ROS, especially in obese women suffering from lipotoxicity, namely intracellular lipid accumulation, inflammatory response, and endoplasmic reticulum stress [25]. On the other hand, an appropriate intake of proteins, antioxidants, and methyl-donor supplements (1-Carbon Cycle) may decrease the bioavailability of ROS [26].

## 4. Oocyte Mitochondria: Biogenesis, Structure, Distribution, and Role in the Acquisition and Maintenance of Oocyte Competence

**The structure of oocytes mitochondria.** The structure of oocyte mitochondria is unique: they are round, rather than being rod-shaped, and the inner membrane is smoother with few, truncated cristae, indicative of a reduced OXPHOS activity [27]. During the pre-implantation period, mitochondria undergo a specific structural change at the blastocyst stage, associated with the re-initiation of mitochondrial biogenesis, which re-establishes the typical form they have in somatic cells. Accordingly, a sharp increase in glycolysis and oxygen consumption characterizes the peri-implantation embryonic stage [28]. 

There is a clear relationship between mitochondria morphology and their functioning, a phenomenon imputable to the absence of specific mitochondrial proteins and transcription factors [29]. Similarly, mitochondrial swelling (a consequence of the loss of the homeostatic control of their volume) and cristae disruption are often observed in oocytes from advanced maternal age women and seem to contribute to the impairment of their bioenergetic metabolism [30].

**Localization of mitochondria within the oocytes.** Mitochondria localization and density during oogenesis and embryogenesis is regulated to meet the changing needs of the cell(s). They generally distribute in areas of increased ATP requirements, although the patterns of mitochondrial distribution in mature oocytes differ among species [15,31,32,33]. In mice, the mitochondria are homogeneously distributed within the ooplasm of GV oocytes, while they re-distribute in MI oocytes to aggregate with the endoplasmic reticulum around the spindle, which is coherent with the increased need for ATP production in order to reach the MII stage. Finally, the mitochondria re-distribute in MII oocytes, again throughout the ooplasm, to be ready for the fertilization process [34]. In humans, the mitochondria of primordial follicles show a perinuclear distribution, whereas their distribution is peripheral in small antral follicles. In large GV oocytes, again, their distribution is perinuclear, therefore they are excluded from the cortical cytoplasm. In MII oocytes and in zygotes, mitochondria are instead distributed throughout the ooplasm, especially in the sub-plasmalemmal and peri-cortical cytoplasm [29].

In summary, whether their distribution is peripheral or perinuclear, mitochondria often show forms of compartmentalization and micro-zonation based on their function, for instance, providing energy for chromosomal segregation and cell division if close to the spindle and participating in Ca^2+^ cycling if close to the endoplasmic reticulum. Therefore, their location might be associated with successful fertilization and embryogenesis downstream [15,29].

**Mitochondrial biogenesis.** Primordial germ cells face a reduction in the number of mitochondria, known as the “bottleneck effect”, which is possibly meant to preserve mitochondria integrity by safeguarding only a few [35]. The mitochondria passing through this bottleneck process will gradually increase in number during oogenesis until they reach >100,000 at the MII stage [36]. No further replication of mitochondria occurs thereafter, thus their initial amount must be divided within the blastomeres during embryo division and be sufficient to support embryo development from fertilization up to the peri-implantation stages [37]. This whole process of growth and division of pre-existing mitochondria is known as mitochondrial biogenesis [38]. Along with whole mitochondria, the copies of mtDNA also seem to follow the same reduction and increase process [35]. Mitochondrial biogenesis is then re-activated at compaction or cavitation [27,29], immediately before blastulation, when the existing mitochondria and their mtDNA are finally amplified to supply the embryo with both energy and oxygen, critical for protein synthesis and for the activation of the sodium pumps involved in filling the blastocoel cavity with the blastocoel fluid [39]. This intensive metabolic activity, in fact, concerns mainly the trophectoderm rather than the inner cell mass, which instead seems quieter and contains less mitochondria [39]

A premature mitochondrial biogenesis before compaction should be considered an atypical phenomenon, possibly indicative of low mitochondrial content or poor mitochondrial activity, and thus of impaired embryonic homeostasis. In fact, this phenomenon associates with hypofunctional mitochondria, mtDNA copies <200,000, increased mtDNA mutation rate, reduced ovarian reserve, and advanced maternal age. In other terms, it is indicative of a compensatory mechanism and therefore suggestive of compromised embryo quality [40,41]. The whole process of mitochondrial biogenesis is subject to environmental influence linked to exercise, caloric restriction, low temperature, oxidative stress, cell division, renewal, and differentiation [38]. 

**The quiet embryo hypothesis.** The “quiet embryo hypothesis” postulates that viable embryos have a “quieter” metabolism than developmentally incompetent ones [42], and the evidence that the mitochondrial activity of early preimplantation embryo is “turned down” at a minimum functional level is in line with this hypothesis. One of the possible explanations for this is limiting the production of ROS, while providing a minimum energy level to sustain early development. In this regard, the main energy substrates are pyruvate, lactate, and aspartate, since early embryos have a limited capacity to use glucose, mainly due to the inhibition of the phosphofructokinase, the enzyme responsible for glycolysis [28]. Similarly, all the DNA replication and cell division events occur with no or limited increase in cellular volume and biomass [43]. 

**Maternal Nutritional Intake and Mitochondrial Activity: Implications for Fertility.** Unbalanced nutritional intakes have been associated with infertility and scarce outcomes after MAR [44]. For instance, MII oocytes collected from obese women tend to be smaller and display a higher frequency of spindle abnormalities, ultimately resulting in increased chromosomal errors when compared to patients with normal body mass index [45,46]. Additionally, an excess of free fatty acids in the follicular fluid associates with abnormal cumulus–oocyte complex morphology [47]. However, studies using human oocytes are scarce and limited, especially since they are mainly conducted on immature or unfertilized oocytes. Therefore, the data in our possess were mainly based on animal models like murine oocytes, and are only indicative of a potential similar scenario in humans. However, they certainly represent alerts that cannot be overlooked. 

Hereafter, we summarized the impact of different unbalanced dietary intakes on the oocytes caused by defective mitochondrial activity. 

## 5. High-Fat (HF) Diet 

**HF diet and mitochondrial distribution.** Maternal obesity prior to conception alters mitochondrial activity in mice starting from their distribution [48]. Instead of revealing a uniform localization, Igosheva and colleagues reported clusters of mitochondria in the proximity of cortical and perinuclear regions in the oocytes collected from obese mice. This evidence was also echoed by Hou et al [49], which is indicative of metabolic/functional damage, in turn involving a greater need for energy production from the oocyte to attempt at preserving its viability and competence [15]

**HF diet and mitochondrial structure.** The ultrastructure of oocyte mitochondria is altered by a HF diet in mice [49,50,51]. Specifically, some authors have described (i) fewer and disarrayed cristae, (ii) more intra-organelle vacuoles, (iii) decreased electron density of the matrix, and (iv) abnormal swelling. Boudoures and colleagues correlated the altered shape of the oocyte dumbbell-like mitochondria to disturbances of their dynamics, suggesting the occurrence of fission and fusion. Remarkably, they observed a significant increase in the so-called “rose petal” mitochondria, which are caused by deficiencies in the oligomerization of the F1F0-ATP synthase, the enzyme responsible for ATP production in the mitochondrial inner membrane. However, a normal morphology might be restored by physical exercise, while the presence of rose petal mitochondria is chronic in sedentary mice exposed to a HF diet [51]. This evidence is intriguing since it suggests that the adverse effects of an unbalanced diet can be partially compensated by a healthy lifestyle. 

**HF diet affects mitochondrial function and leads to oxidative stress.** Igosheva and colleagues reported that the excessive supply of metabolic substrates in mice results in a significant increase in inner membrane potential, mitochondrial respiratory activity, and oxidation processes [48]. Conversely, Wu et al. reported a reduction in the inner membrane potential, possibly related to the release of Ca^2+^ from the endoplasmic reticulum in mice affected by Alstrom syndrome (a condition leading to severe obesity) [52]. In general though, several groups are concordant in reporting an alteration of the mitochondrial membrane potential and increased production of ROS, in turn triggering oxidative stress in the oocytes collected from obese mice [48,49,50,52]. An involvement in the endoplasmic reticulum was also consistently reported by another group in 2017, which showed increased levels of mitochondria-associated endoplasmic reticulum membrane proteins (MAMs), increased levels of Ca^2+^, higher risk for apoptosis, and compromised cytoplasmic maturation in the oocytes collected from obese women [53]. When it comes to energy availability, these mitochondrial impairments can potentially lead to defects in citric acid cycle metabolism and hydroxyacyl-CoA dehydrogenase (HADHA) functioning (a subunit of an enzyme critical for fatty acid β-oxidation), however, it is unclear whether this affects ATP production [50,51].

**HF diet leads to premature mitochondrial biogenesis.** The functional defects in the existing mitochondria might be compensated with an increase in mtDNA replication and by initiating mitochondrial biogenesis prematurely, which is a phenomenon reported by both Igosheva’s and Luzzo’s groups [48,50].

**HF diet affects meiotic spindle integrity.** Spindle abnormalities and chromosome misalignment have been suggested among the possible consequences of a HF diet. Although it is not clear whether this directly involves altered mitochondria, it surely results in a higher prevalence of chromosomal missegregation events [50]. 

All these mitochondrial defects are associated with lower fertilization, higher arrest at EGA, and lower blastulation in fertilized oocytes retrieved from obese mice [48,50,52] 

## 6. High-Fat and High-Sugar (HF/HS) Diet 

**HF/HS diet affects mitochondrial structure and distribution.** A HF/HS diet in mice results in larger mitochondria, a modification of their typical round-shaped morphology, with irregular vacuoles enclosing lamellar membranes [54]. Remarkably, these mitochondrial defects can be trans-generational and affect up to the F3 generation of these mice, possibly leading to mitochondrial dysfunction throughout the whole organism [54]. In fact, altered mitochondrial distribution, reduced mitochondrial mass, and decreased ATP and citrate levels also persist in the oocytes of the following generations [55]. Presumably, there is an epigenetic effect deriving from a HF/HS diet, thus further studies are needed to fully define the mechanisms eventually implicated.

**HF/HS diet affects mitochondrial function, spindle integrity and leads to increased ROS levels.** A HF/HS diet causes a re-distribution of the mitochondria and a consequent modification of their function in the oocytes [56]. Accordingly, decreased levels of ATP, citrate, and creatine phosphate, and higher levels of ROS were reported. Moreover, an impairment of the meiotic spindle was revealed, suggesting an implication of defective mitochondria in the process of chromosomal segregation. Remarkably, when Boots and colleagues administrated a CoQ10 antioxidant to the mice fed with a HF/HS diet, the ROS levels in the oocytes decreased, and spindle organization and chromosome alignment were re-established. 

## 7. Low Protein (LP) Diet 

**LP diet affects mitochondrial structure, biogenesis and function, and leads to pro-oxidant imbalance.** Although the evidence is limited to a single study, protein limitation (with or without folate supplementation) might affect mitochondrial ultrastructure. This evidence has been reported by Schutt and colleagues in the cumulus oocyte complexes in murine models [57]. They also reported an impact of LP diet on the expression of Drp1, Opa-1, Mfn1/2, Parl, and Ndufb6, which might lead to defective mitochondrial biogenesis, and misregulation of apoptosis, respiratory electron transport, and glucose metabolism in the oocytes. Even in this case, the supplementation of folate was ineffective and the correct level of gene transcription could not be restored. Finally, Schutt and colleagues highlighted aberrant metabolic turnover pathways in the oocytes retrieved after protein starvation. In particular, serine turnover reduction resulted in diminished trans-sulfuration and increased oxidative stress since ROS could not be contrasted. Moreover, serine provides the methyl groups required for metabolic pathways and DNA, RNA, and protein production. Thus, it might be hypothesized as an effect of maternal protein starvation on oocyte competence downstream. Interestingly, folic acid supplementation in this case allowed a partial normalization of serine turnover levels in the oocytes. 

## 8. The Effect of Unbalance Nutritional Intake on Oocyte Mitochondrial Activity: Seeing the Big Picture

**Figure 1:**
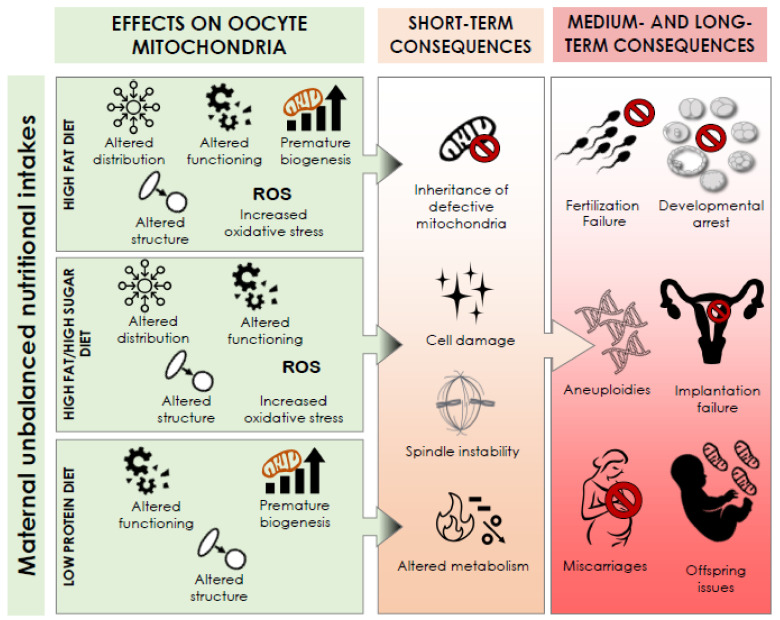
Summary of the effects of maternal unbalanced nutritional intake on oocyte mitochondria and their putative short-, medium-, and long-term consequences.

This review summarizes the present body of evidence that supports a possible impact of maternal nutritional intake on oocyte competence via a defective mitochondrial biogenesis, structure, distribution, and function in mice (Table 1 and Figure 1). 

Nutrients, just like pollutants, can reach any tissue in our body via the bloodstream, and be eventually delivered in the follicular fluid [58,59], which is presumably their main route of entrance in the oocyte. In 2012, Leese underlined that maternal dietary intake is the main source of nourishment for the oocyte and the early embryo [60]. Therefore, unbalanced supplies represent an important source of stress for the female gametes. Mitochondria, as powerhouses of the cell, play a critical role in this delicate metabolic balance. If impaired, they might have serious consequences on oocyte competence until EGA and beyond [6,30,61]. The main reason for this is an insufficient ATP production, in turn undermining nuclear maturation, polymerization of spindle microtubules, chromosome segregation, membrane biosynthesis, and cell division [62]. Moreover, the increase of ROS due to inefficient mitochondrial functioning certainly does not represent the ideal environment for the fertilization and developmental processes. In fact, it has been underlined that a reduction in oocyte developmental competence by stress is associated with alterations in mitochondrial function [63]. 

Investigating the association between obesity (or high body mass index levels in general) and infertility is straightforward [64,65], however, it also issues an alert toward a more subtle and detailed analysis of the cause-and-effect relationship between HF and HF/HS diets and oocyte competence. Certainly, the increased presence of adipose tissue is the key, since the correlation between adipose tissue and lipid toxicity, inflammation and ROS production has been widely reported [1,66,67]. This inflammation is imputable to the oxidative stress generated by mitochondria due to an excessive oxidation of their substrates, both pyruvate and fatty acids. On one hand, the over-intake of sugars can lead to extensive glycolysis and excessive pyruvate oxidation, in turn causing electron leakage from the electron transport chain and partial reduction of O_2_ to O_2_^−^ generating ROS; on the other hand, instead, it has been demonstrated that lipid accumulation in non-adipose tissue also induces lipotoxicity and oxidative stress due to the excess of elevated circulating triglycerides and free fatty acids. As a consequence, the excess of free fatty acids remains in the cytosol and may undergo lipid peroxidation, generating ROS [68]. Furthermore, free fatty acids may also indirectly induce oxidative stress by affecting the endoplasmic reticulum and increasing the intracellular level of Ca^2+^, which then enters the mitochondria and increases ROS production via higher pyruvate dehydrogenase activity, the enzyme responsible for the transport and oxidation of pyruvate into acetyl CoA [68]. Most likely, all these features contribute to a decreased oocyte quality, reduced blastulation, increased risk for miscarriage, and reduced live birth rate after MAR in obese patients [64,65]. 

The effect of protein restriction has been less investigated, but this unbalanced maternal nutritional intake might also be responsible for significant effects on fertility even in the following generations, as reported in animal models [69,70,71,72]. Although protein restriction in somatic cells results in a metabolic shift to produce antioxidant precursors and detoxify the ROS produced by altered amino acid metabolism in the mitochondria, this does not occur in the oocytes with obvious detrimental consequences. However, folicacid supplementation seems to help limit some of these consequences [57]. 

This manuscript is structured as a narrative non-systematic review, although it has been conducted to comprehensively cover the current knowledge on the potential impact of unbalanced nutritional intake on oocyte mitochondria and on their reproductive consequences. All the original papers published to date are based on the murine model, and if on one hand this information cannot be directly translated in humans, on the other hand the well-controlled experimental conditions help limit all putative sources of bias on the outcome.

## 9. Therapeutic Future Perspectives

Restoring or improving mitochondrial function and/or content might be helpful to counteract infertility. Different studies in the literature have described the strategies under investigation to this end. The administration of rapamycin or resveratrol, antioxidant therapy, and mitochondrial transfer figure among them [35]. While the investigation of pharmaceutical therapies is mainly based on assessing their efficacy, mitochondrial manipulation instead also raises ethical issues. In fact, this latter approach entails the supplementation of an oocyte with additional mitochondria isolated from a younger donor, thereby involving mitochondrial heteroplasmy, namely the coexistence of two mtDNA genomes in the same embryo. To overcome this issue, some authors have tried to explore whether autologous mitochondria extracted from somatic cells can be used as a source of additional mitochondria to inject into the inseminated oocyte [73,74]. Nevertheless, the clinical results to date are far from promising [75]. Additionally, the treatment of oocytes with β-oxidation inhibitors has been proposed as a solution for inhibiting the utilization of free fatty acids as a substrate and limiting ROS production, inducing a shift to carbohydrate metabolism [76,77,78,79,80,81]. However, in this case, an over-compensatory shift also resulted in the production of ROS [82]. In other terms, a balance is required between the oxidation of pyruvate and fatty acids by mitochondria to preserve the cellular homeostasis, and ultimately, oocyte competence.

Finally, the use of antioxidants has also been reported to counteract the detrimental effect of oxidative stress on cell function (reviewed by [83]). However, although on the one hand, antioxidants can be useful for protecting the oocytes from the damaging effects of ROS, on the other hand, physiological levels of ROS play a key role in different cellular processes such as oocyte maturation, ovarian steroidogenesis, corpus luteal function, and luteolysis [19]. Therefore, the utilization of antioxidants should be modulated carefully. Up to now, antioxidant supplementation does not appear to offer any benefit to women with female subfertility [84] or undergoing infertility treatment [83,85,86].

Certainly, education is rather advisable in the future aimed at improving the nutritional balance between the maternal intake of sugar, proteins, and fats, especially paying particular attention to the typology of fatty acids. Omega-3 polyunsaturated fatty acids (PUFA), acting as natural antioxidants [87], are preferable to trans-fatty acids before, during, and after the peri-conceptional period, which has been demonstrated to positively associate with the probability of achieving a live birth among women undergoing ART [88] and, in general, for enhancing female fertility [89]. This is critical to avoid molecular and endocrinological problems such as insulin resistance, inflammation, hypertension, cardiovascular, and coagulation issues [64], and possibly also improve the long-term reproductive prognosis [90].

## Figures and Tables

**Table 1:** Summary of the effects of unbalanced nutritional intake on oocyte mitochondria. All articles were original investigations conducted on the murine model. HF, High Fat; HF/HS, High Fat/High Sugar; LP, Low Protein.

Paper	Unbalanced Nutritional Intake	Effects on Mitochondria				
		Altered Distribution	Altered Structure	Altered Functioning	Increased ROS	Altered Biogenesis
Andreas et al., 2019 [55]	HF/HS	X	X	X		
Boots et al., 2016 [56]	HF/HS	X	X	X	X	
Boudoures et al., 2016 [51]	HF		X			
Hou et al., 2016 [49]	HF	X	X			
Igosheva et al., 2010 [48]	HF	X		X	X	X
Luzzo et al., 2012 [50]	HF		X			X
Saben et al., 2016 [54]	HF/HS		X	X		
Schutt et al., 2019 [57]	LP		X	X	X	X
Wu et al., 2015 [52]	HF			X	X	
Zhao et al., 2017 [53]	HF			X		
